# *Panstrongylus rufotuberculatus* (Champion, 1899) (Hemiptera, Reduviidae, Triatominae) in Rondônia, Brazil: A novel report

**DOI:** 10.1590/0037-8682-0141-2021

**Published:** 2021-08-20

**Authors:** Dayse da Silva Rocha, Alda Lobato, Cleber Galvão

**Affiliations:** 1 Fundação Oswaldo Cruz, Instituto Oswaldo Cruz, Laboratório Nacional e Internacional de Referência em Taxonomia de Triatomíneos, Rio de Janeiro, RJ, Brasil.; 2 Laboratório Central de Saúde Pública, LACEN, Porto Velho, RO, Brasil.

**Keywords:** Chagas disease, New records, Vectors, Surveillance programs

## Abstract

**INTRODUCTION:**

This short communication presents a novel report on the occurrence of *Panstrongylus rufotuberculatus* in the Brazilian state of Rondônia.

**METHODS:**

Two specimens were collected inside dwellings and identified using dichotomous keys.

**RESULTS:**

The present study showed the extensive geographic distribution of *P. rufotuberculatus* and the increased number of species in the state of Rondônia.

**CONCLUSIONS:**

This new record of *P. rufotuberculatus* is important for understanding the epidemiology of Chagas disease because this species is found naturally infected with *Trypanosoma cruzi*. Studies on the ecology, biology, and vector-host-parasite interactions of this species are essential for surveillance programs.

Blood-sucking insects of the subfamily Triatominae (Hemiptera: Reduviidae) include 153 extant and three fossil species assigned to five tribes and 18 genera, all the extant species being considered potential vectors of *Trypanosoma cruzi* (Chagas, 1909), the etiologic agent of Chagas disease^1^ ^,^ ^2^. Among the five tribes, Triatomini is the most diverse, with more than 70% of the species of the subfamily. The genus *Triatoma* is the most speciose within the tribe (82 species), followed by *Panstrongylus* (15 species). The tribe has the widest geographical distribution among Triatominae, reaching an extensive range of ecotopes^3^.

*Panstrongylus rufotuberculatus* (Champion, 1899) is a wild species widely distributed in South America; its occurrence has been reported in the Brazilian states of Acre; Amazonas; Mato Grosso; Pará; and the neighboring countries of Argentina, Bolivia, Colombia, Costa Rica, Ecuador, French Guayana, Peru, Suriname, and Venezuela. In Central and North America, it has been found only in Panama and Mexico (Figure 1A)^3^ ^,^ ^4^. 

**FIGURE 1:**
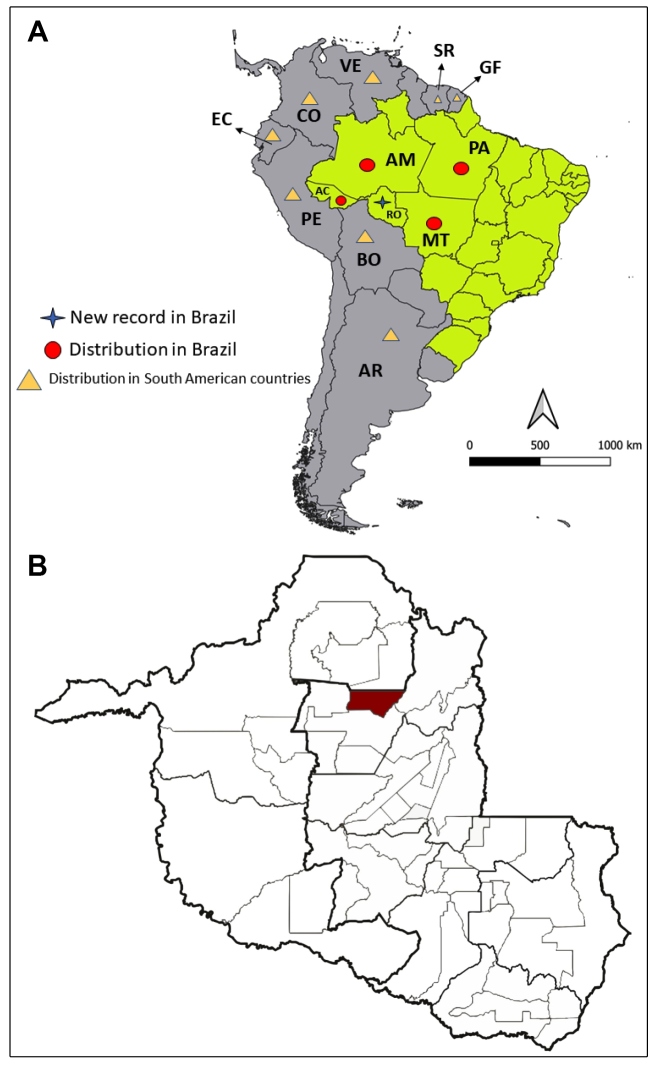
**(A)** Map of South America showing the previous, and the new record of *Panstrongylus rufotuberculatus* (Champion, 1899) by Brazilian states and South American countries. **(B)** Location of Rio Crespo, RO, Brazil, where two specimens of *Panstrongylus rufotuberculatus* (Champion, 1899) were collected.

Despite being reported a long time ago, the literature on *P. rufotuberculatus* is scarce, and it remains among the least known triatomines. The first report of natural infection caused by *T. cruzi* was published in Venezuela in 1940, and since then, its natural infection has been reported in Argentina, Bolivia, Costa Rica, Colombia, Ecuador, and Peru^6^ ^,^ ^9^ ^,^ ^10^. In Argentina, specimens of *P. rufotuberculatus* were found in dwellings and attributed to the probable “attraction” to the ligths^5^. Incipient domiciliation has also been reported in Bolivia and Ecuador^7^ ^,^ ^8^. However, authors have already reported intradomiciliary colonies of *P. rufotuberculatus* in Peru, where several nymphs and adults were collected inside the dwellings^9^ ^,^ ^10^. In Colombia, *P. rufotuberculatus* is considered a species with a high epidemiological risk for the transmission of *T. cruzi*, constituting the second most common triatomine caught inside dwellings^11^.

In August 2018, a male specimen of triatomine was found inside a dwelling by the resident in the municipality of Rio Crespo (latitude 09^o^ 42’18” S and longitude 62^o^ 53’59” W), Rondônia state, Brazil (Figure 1B). This insect, *Panstrongylus rufotuberculatus*, was identified by the third author (CG) based on their external morphological characteristics through photographic records. For the identification of the genus *Panstrongylus,* the main criterion is the position of the antennae, inserted close to the eyes. The diagnosis of the species is based mainly on jugae blunt, tubercles of the fore lobe of pronotum invariably red, overall color of hemelytra pale green^3^ (Figure 2 and Figure 3). Two years later (October 2020), another male specimen was caught in the same municipality in a dwelling 10 km away from the first dwelling. Both specimens were caught inside dwellings by the residents and handed over to the endemic agents who referred them to Laboratório Central de Saúde Pública (LACEN-RO). The second specimen (Figure 2) was sent to the Laboratório Nacional e Internacional de Referência em Taxonomia de Triatomíneos, Oswaldo Cruz Institute, Fiocruz, Rio de Janeiro, where it was identified as *P. rufotuberculatus*, through the dichotomic keys^3^. The voucher specimen was deposited at the Herman Lent Collection of Triatominae Collection of the Oswaldo Cruz Institute (CTIOC) (number HL 3458). Natural infection caused by *T. cruzi* in the insects was analyzed by abdominal compression, and the contents were evaluated by direct microscopic examination; both specimens were negative.

**FIGURE 2:**
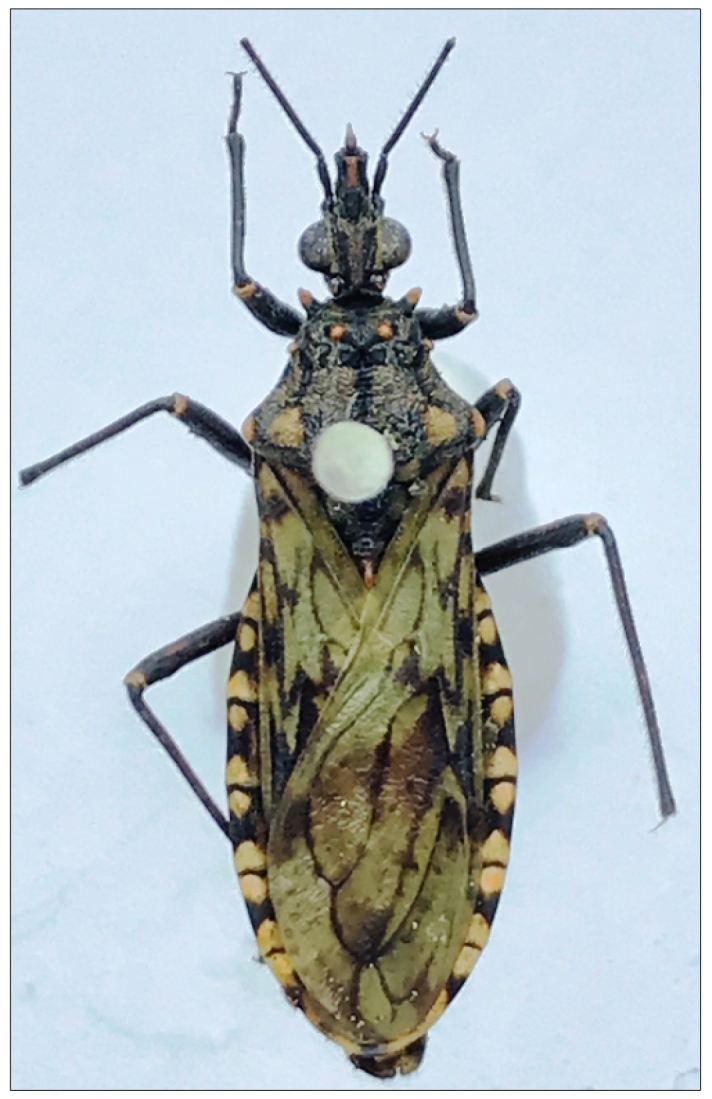
Dorsal view of a male *Panstrongylus rufotuberculatus* (Champion, 1899), with a total length of 24 mm. The tubercles of the fore lobe of pronotum are invariably red, an important diagnostic character.

**FIGURE 3:**
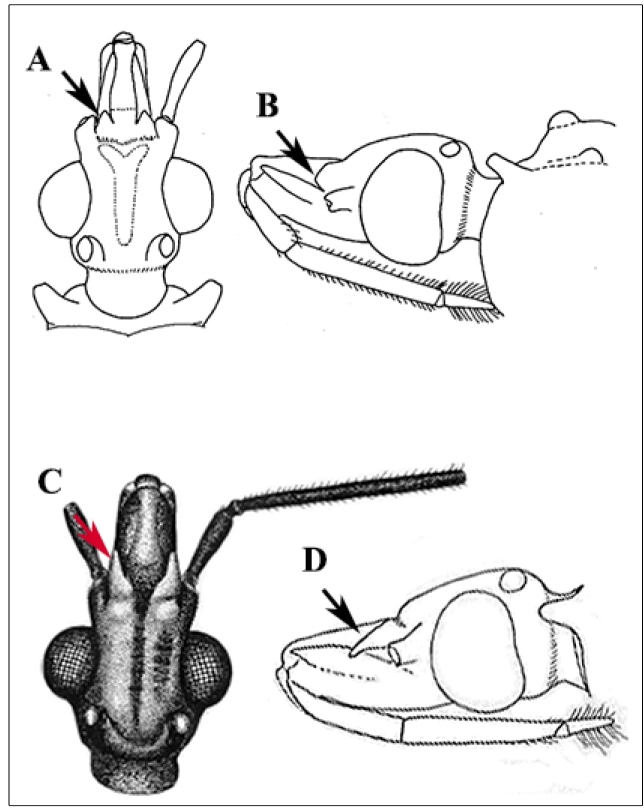
**(A, B)** Arrows indicate the jugae blunt, another important diagnostic characteristic of *Panstrongylus rufotuberculatus* **(**Champion, 1899). **(B, C)** Jugae sharped, *Panstrongylus* spp.

The present report demonstrates that nine species of triatomines were found in the state of Rondônia: *Eratyrus mucronatus* Stål, 1859, *Panstrongylus geniculatus* (Latreille, 1811), *P. lignarius* (Walker, 1873), *P. megistus* (Burmeister, 1835), *P. rufotuberculatus* (Champion, 1899), *Rhodnius milesi* Carcavallo, Rocha, Galvão, and Jurberg, 2001, *R. montenegrensis* Rosa *et al.*, 2012, *R. pictipes* Stål, 1872, and *R. robustus* Larrousse, 1927^3^ ^,^ ^12^ ^,^ ^13^ ^,^ ^14^ ^,^ ^15^. The data of the geographical distribution of *P. rufotuberculatus* to Rondônia state are consistent with those in the prediction map for the potential distribution of this species in Brazil presented by Galvão^3^, but these findings in North Brazil are limited to a few specimens^3^. Knowledge of the biology of this species is scarce, and in natural environments, this species has been found to feed on bats, armadillos, domestic animals, and humans, but its habits are usually wild^3^ ^,^ ^4^ ^,^ ^7^. 

The increase in the number of reports of *P. rufotuberculatus* domiciliation may be related to changes in the wild environment, causing displacement to domestic environments as an alternative in the search for food. This finding emphasizes the need for careful entomological and epidemiological surveillance of this and other triatomine species in the Amazon region.
